# Silicon-Deposited Porous Carbon Anodes: A Design Paradigm for Next-Generation High-Energy-Density Lithium-Ion Batteries

**DOI:** 10.1021/acs.nanolett.6c03725

**Published:** 2026-09-28

**Authors:** Guifang Zeng, Jing Li, Hamid Mollania, Karol Viviana Mejia-Centeno, Malik Dilshad Khan, Yanhong Tian, Andreu Cabot, Qing Sun

**Affiliations:** † State Key Laboratory of Precision Welding and Joining of Materials and Structures, 47822 Harbin Institute of Technology, Harbin 150001, China; ‡ Catalonia Institute for Energy Research (IREC), Sant Adrià de Besòs, Barcelona 08930, Spain; § Department of Electronic and Biomedical Engineering, University of Barcelona, Barcelona 08028, Spain; ∥ Faculty of Chemistry, University of Barcelona, Barcelona 08028, Spain; ⊥ ICREA, Pg. Lluis Companys, Barcelona 08010, Spain

**Keywords:** Silicon anodes, Silicon-deposited porous carbon, Silane, Solid electrolyte interphase, Lithium-ion batteries

## Abstract

Silicon is a promising anode for high-energy-density lithium-ion batteries (LIBs) due to its exceptionally high theoretical capacity, yet severe volume expansion and interfacial instability hinder practical application. Silicon-deposited porous carbon (SiDPC), which spatially confines nanosized silicon within porous carbon frameworks, offers a distinct structural paradigm beyond conventional mechanically blended Si–C composites. This mini-review summarizes the evolution and engineering principles of SiDPC architectures, highlighting how structural regulation governs electrochemical performance. Particular attention is given to the synergistic management of volume expansion, stress evolution, electron/Li^+^ transport, and solid electrolyte interphase formation, through which SiDPC has evolved from passive buffering to active structural regulation. Remaining challenges in scalable synthesis, electrode engineering, and practical application are discussed, alongside opportunities for programmable Si–C architectures enabled by intelligent structural design. This review provides mechanistic insights and design guidelines for advancing SiDPC toward practical high-energy-density silicon anodes for LIBs.

## Graphical abstract

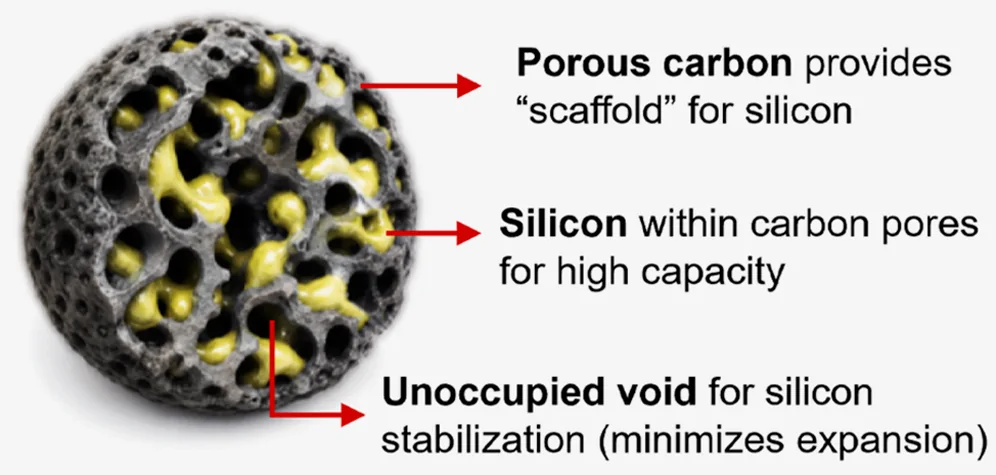

## 1 Introduction

Driven by the accelerating demand for electrification and high-energy-density energy storage, lithium-ion battery (LIB) anodes are undergoing a fundamental transition from graphite toward silicon, establishing silicon as the central focus of next-generation anode development.^1^ Its exceptionally high theoretical specific capacity (∼3579 mAh g^–1^) underpins this transition.^2^ However, its practical deployment remains constrained by > 300% volume expansion,^3,4^ intrinsically low electronic conductivity,^5^ and the formation of an unstable solid electrolyte interphase (SEI).^6^

Silicon anodes have evolved from nanostructure engineering to composite architecture design.^7^ Early efforts focused on nanosized silicon to alleviate volume expansion through size reduction and structural buffering (Figure 1). However, its high specific surface area intensified parasitic reactions and irreversible capacity loss.^8^ Silicon–carbon (Si–C) composites subsequently emerged as a promising strategy, where conductive carbon frameworks enhance electron transport while regulating stress distribution and SEI formation,^9^ yet precise control over interfacial chemistry and long-term SEI stability remains elusive.^10^ Silicon oxide (SiO_*x*_) has since attracted considerable attention owing to its intrinsic self-buffering capability,^11,12^ enabling the in situ formation of buffering phases (e.g., Li_2_O and Li_4_SiO_4_) during cycling.^13–15^ Nevertheless, its intrinsically low electronic conductivity and complex reaction pathways continue to compromise rate capability and energy efficiency.

**Figure 1:**
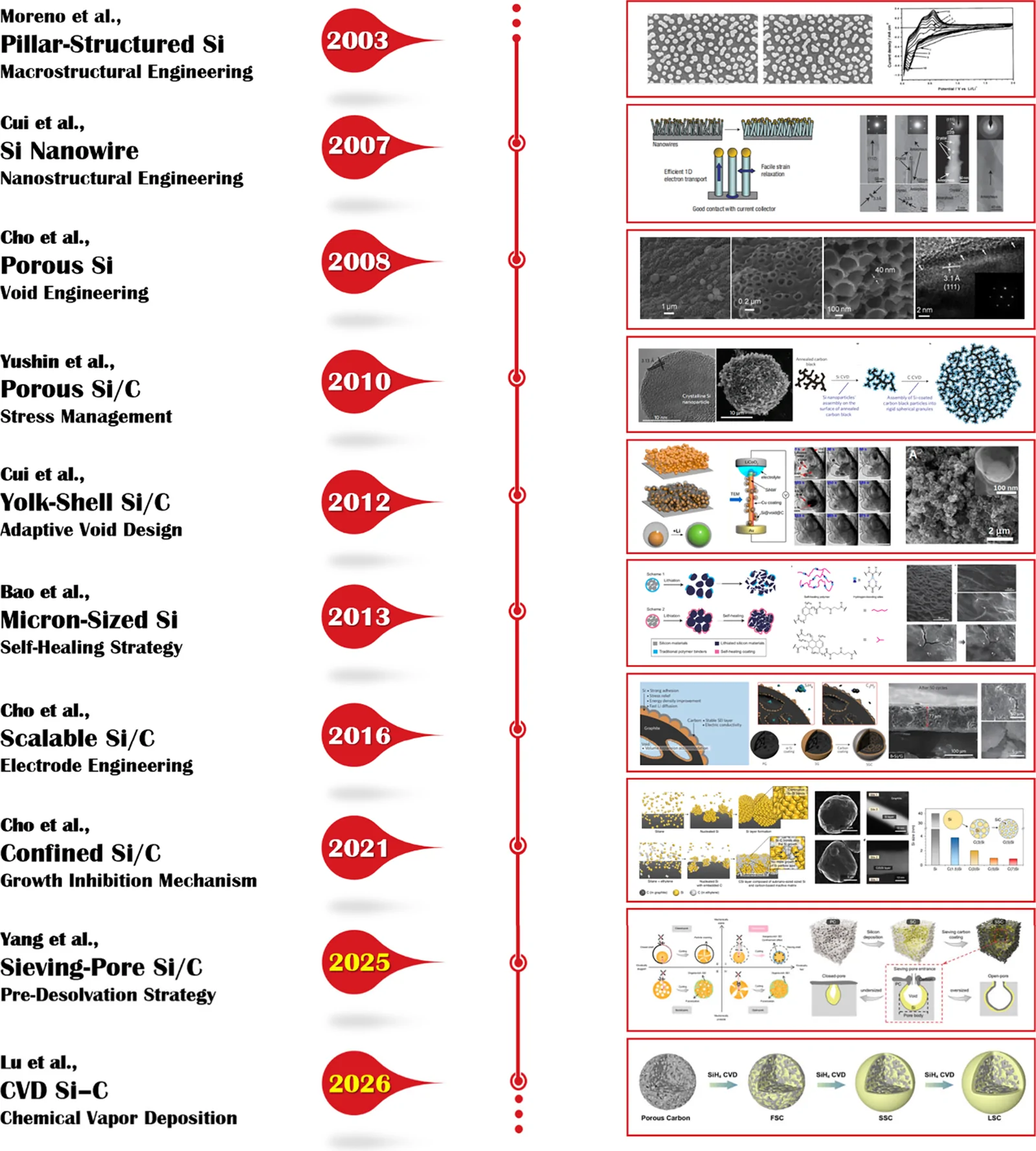
Development history and major milestones of silicon-based anodes. Reproduced with permission from ref 43. Copyright 2003 IOP Publishing. Reproduced with permission from ref 44. Copyright 2007 Springer Nature. Reproduced with permission from ref 45. Copyright 2008 Wiley. Reproduced with permission from ref 46. Copyright 2010 Springer Nature. Reproduced with permission from ref 18. Copyright 2012 American Chemical Society. Reproduced with permission from ref 47. Copyright 2013 Springer Nature. Reproduced with permission from ref 26. Copyright 2016 Springer Nature. Reproduced with permission from ref 24. Copyright 2021 Springer Nature. Reproduced with permission from ref 23. Copyright 2025 Springer Nature. Reproduced with permission from ref 29. Copyright 2026 American Chemical Society.

More recently, silicon-deposited porous carbon (SiDPC) has emerged as a promising structural paradigm inspired by SiO_*x*_-based architectures,^27^ in which nanosized silicon is confined within porous carbon frameworks (Figure 1). This architecture has attracted growing interest as a practical route toward high-energy-density silicon anodes, particularly with the development of microsized chemical vapor deposition (CVD)-derived Si–C materials. Nevertheless, SiDPC is frequently used interchangeably with Si–C architectures, although these materials can differ fundamentally in their preparation philosophy, silicon distribution, and pore architecture. However, these materials differ fundamentally in their preparation philosophy, silicon distribution, and pore architecture. As summarized in Table 1, SiDPC should be regarded as a distinct structural category characterized by the deposition of silicon within a preformed porous carbon framework, in which the internal pore network is deliberately preserved to accommodate silicon volume expansion and regulate interfacial evolution. This structural configuration distinguishes SiDPC from conventional mechanically blended Si–C composites, carbon-coated Si, and other porous Si–C architectures, where silicon is typically dispersed throughout the carbon matrix or encapsulated by a carbon layer.

**Table 1:** Structural Classification of Si–C Anode Architectures and the Scope of SiDPC.^16–26^

architecture	preparation principle	Si distribution	void design	distinguishing feature	cost
mechanically blended Si–C	physical mixing	between carbon particles	uncontrolled	conventional composite	low
carbon-coated Si	carbon formed around Si	Si core	partial voids possible	core–shell	low-moderate
porous Si–C composite	various routes	dispersed in carbon	variable	broad category	low-moderate
SiDPC	Si deposited within preformed porous carbon	confined within internal pore network	deliberately retained	distinct structural paradigm	moderate-high
Si-deposited graphite	CVD in porous graphite	internal and/or external	depends on graphite structure	special case	moderate

Mechanically blended Si–C and carbon-coated Si are generally advantageous in terms of simple processing, low cost, and scalability, but provide limited control over silicon confinement and volume expansion. More sophisticated porous and core–shell/yolk–shell architectures offer greater free volume and improved structural stability,^28^ but often require more complex fabrication, resulting in higher costs, lower tap density, and challenges in large-scale production. SiDPC provides a balanced structural design by combining a preformed porous carbon scaffold with controlled silicon deposition, enabling confined silicon growth, effective volume accommodation, and improved interfacial stability. Although CVD-based Si deposition may involve relatively high processing temperatures, energy consumption, and precursor costs, its compatibility with scalable gas-phase processing and tunable Si loading makes SiDPC a promising architecture for high-performance and practically relevant Si anodes. These trade-offs highlight that the optimal Si–C architecture should be evaluated not only by electrochemical performance but also by fabrication complexity, cost, and scalability.

By integrating silicon into a pre-engineered porous carbon scaffold, this strategy enables precise regulation of silicon distribution, pore architecture, and interfacial characteristics, thereby effectively accommodating volume variation, stabilizing the SEI, and facilitating electron/ion transport.^38^ Engineering-scale implementations, first exemplified by developments from Group14 Technologies (G14),^41^ have further demonstrated the practical viability of this material platform,^42^ establishing a representative route toward the commercialization of high-energy-density silicon anodes. Subsequent studies have further expanded the SiDPC family through diverse synthesis strategies and structural designs, and representative examples together with their electrochemical performance are summarized in Table 2.

**Table 2:** Electrochemical Performance of Representative SiDPC Anodes

preparation method	deposition conditions	carbon scaffold	cell type	Si loading (mg cm^–2^)	silicon content (wt %)	ICE (%)	current density/C-rate	cycle number	cycling performance (mAh g^–1^)	ref
CVD (silane pyrolysis) + surface oxidation + chemical prelithiation	SiH_4_/Ar (500 °C, 30 mL min^–1^/200 mL min^–1^, 10 h); C_2_H_2_ (550 °C, 15 h)	hard carbon; pore volume = 1.0, 0.8, or 0.6 cm^3^ g^–1^	half-cell	2.5	53.6	91.9	0.2 C	250	1502.8	^29^
two-step CVD (SiH_4_ deposition + C_2_H_2_ carbon coating)	Ar/SiH_4_ = 9:1 (400 °C, 360 min); Ar/C_2_H_2_ = 9:1 (600 °C, 60 min)	spherical microporous carbon; pore size < 2 nm; pore volume 0.757 cm^3^ g^–1^	half-cell	/	43	87.83	1 A g^–1^	500	1240.6	^30^
CVD	/	porous carbon scaffold; internal porous 3D carbon framework	half-cell	/	5	/	/	1200	85.4% capacity retention	^31^
CVD (Si deposition + carbon coating)	SiH_4_/Ar (500 °C, 30/200 mL min^–1^, 10 h); 10 vol % C_2_H_2_/Ar (550 °C, 15 h)	hard carbon; pore volume 0.8 cm^3^ g^–1^	half-cell	/	41	88.41	0.2 C	65	1335	^32^
CVD (SiH_4_/C_2_H_4_ codeposition)	SiH_4_/C_2_H_4_ = 10:5 (475 °C, 50 mL min^–1^, 45 min); 10 vol % C_2_H_2_/Ar (550 °C, 15 h); 5 wt % pitch-based carbon coating (900 °C)	spherical natural graphite; C(5)Si layer containing subnano-Si/SiC/a-C dual matrix	half-cell	/	63.1	90.0	/	500	1262.3	^24^
CVD (SiH_4_ deposition) + interfacial SiC synthesis	SiH_4_ (475 °C, 0.02 L min^–1^); SiC (900 °C, 2 h)	graphite-supported open-ended carbon nanotubes (OCNTs); Si nanolayer uniformly deposited on OCNTs	half-cell	/	/	90.8	0.5 C	50	748	^33^
two-step CVD (Si–C layer + carbon coating)	Ar/H_2_ = 95:5, C_2_H_4_/Ar = 10:90 (850 °C, 40 mL min^–1^, 35 min); SiC (1300 °C, 1 h); outer C shell formed by CVD	Si nanoparticle core with SiC/carbon shells	half-cell	/	72.1	88.5	1 A g^–1^	800	980	^34^
one-step CVD (copyrolysis of SiH_4_/C_2_H_4_) + carbon coating	SiH_4_:C_2_H_4_ = 10:5 (500 °C,1 L min^–1^, 14 h); carbon coating (900 °C)	microsized SiC_*x*_ particles with atomic-level Si/C distribution	half-cell	/	∼80	85.9	0.2 C	100	1227	^35^
thermal oxidation + CVD (SiH_4_ deposition)	/	expanded graphite; interconnected giant macropores; Si preferentially distributed within macropores	half-cell	/	17	92.9	0.5 C	50	914	^36^
one-pot CVD + thermal oxidation + carbon coating	/	sacrificial carbon template; hollow shell/tube/sheet structures; Si nanocrystallites homogeneously embedded in β-SiC matrix	half-cell	/	/	89.7	/	100	86% capacity retention	^37^
CVD (SiH_4_ deposition) + carbon coating	SiH4/Ar = 5/95 (430 °C, 90 min); C_2_H_2_ (600 °C, 30 11 min)	micropore-dominated activated spherical carbon	half-cell	/	31.9	74.64	1 C	800	1301	^38^
CVD (SiH_4_ deposition) + carbon coating	/	Si nanocrystallites homogeneously embedded in β-SiC matrix	half-cell	/	6	92	0.5 C	100	96% capacity retention	^26^
fluidized-bed CVD (Si deposition) + carbon coating	SiH_4_ (560 °C, 340 min, 2.5 L min^–1^); C_2_H_2_ (600 °C, 4 L min^–1^, 140 min)	commercial porous carbon, pore volume 0.85 cm^3^ g^–1^	half-cell	/	52.1	90.6	0.5 A g^–1^	100	1329.7	^39^
CVD SiH_4_–C_2_H_2_ codeposition + carbon coating	/	Si deposited inside carbon-activated carbon pores	half-cell	/	∼40	83.8	0.7 C	350	94.4% capacity retention	^40^

While recent reviews have provided comprehensive perspectives on microsized CVD-derived Si–C anodes,^27^ including their structural evolution, interfacial regulation, and manufacturing challenges, a focused framework linking the characteristic architecture of SiDPC to its underlying electrochemical mechanisms remains lacking. Building upon these advances, this mini-review focuses on the structural design principles and underlying mechanisms of SiDPC anodes, with particular emphasis on their synergistic roles in stress regulation, charge transport, and interfacial stabilization, followed by a discussion of the remaining challenges and future perspectives for their practical implementation under realistic electrode conditions. By bridging fundamental mechanisms with structural engineering, this mini-review aims to provide design guidelines for the rational development of high-performance silicon anodes.

## 2 Engineering Principles of SiDPC Architectures

In 2016, G14 introduced an engineering-oriented SiDPC anode material architecture.^48^ Unlike conventional strategies based on mechanical blending or carbon coating, this approach employs high-surface-area amorphous porous carbon as a 3D scaffold, providing interconnected pore channels for subsequent Si infiltration and internal volume accommodation. Through CVD, silicon precursors uniformly infiltrate the carbon pore network and are subsequently converted into nanoscale amorphous silicon via in situ deposition,^49^ yielding a composite architecture consisting of a porous carbon framework, embedded silicon nanoparticles, and reserved internal voids (Figure 2a). The subsequent commercial release of SCC55 (G14’s flagship Si–C composite anode material) in 2022 marked the successful scale-up of this technological platform.^50^

**Figure 2:**
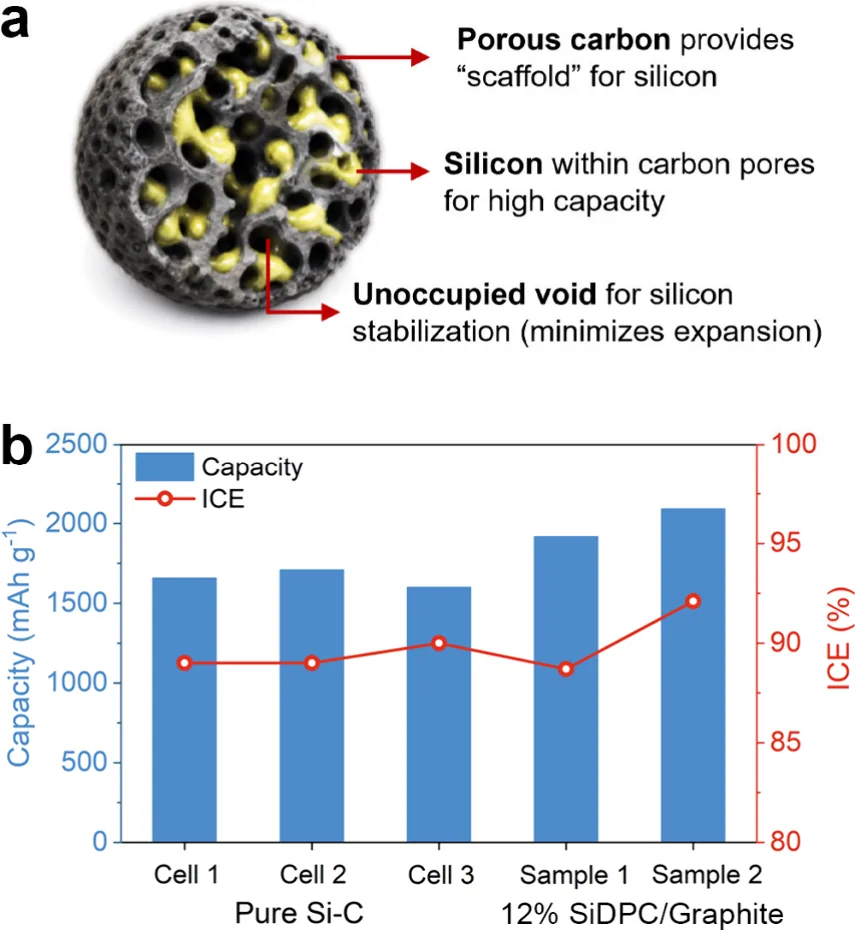
(a) Schematic of G14’s Si–C composite structure. (b) Specific capacity and ICE of Si–C-based anodes from the third-party evaluation.^53^ “Cell” denotes Si–C electrodes, whereas “Sample” denotes electrodes containing 12 wt % SiDPC blended with graphite.

The key feature of this architecture is the confinement of nanoscale silicon within a porous carbon framework containing reserved internal voids, where the pore structure simultaneously regulates Si deposition and accommodates subsequent volume expansion.^51^ This design effectively mitigates volumetric expansion while providing the structural basis for improved mechanical integrity, charge transport, and interfacial stability.^52^ This architecture substantially reduces volume expansion compared with conventional Si–C composites at comparable capacities. In G14’s coin-cell half-cell measurements, in which Si–C was blended with graphite to achieve an anode capacity of approximately 450–650 mAh g^–1^, the Si–C composite exhibited a volume expansion as low as ∼26%, whereas other Si–C composites showed approximately 60–80% expansion at around 600 mAh g^–1^.^53^ This value was comparable to the graphite expansion measured under the same evaluation, highlighting the effectiveness of the confined porous architecture in mitigating electrode expansion.

The choice of carbon scaffold is therefore critical because its pore structure directly governs precursor transport, silicon nucleation, and the available space for volume accommodation. Activated porous carbons provide high surface area and abundant accessible pores, which favor silicon infiltration and high Si loading, but excessive microporosity can restrict precursor diffusion and lead to pore blockage during CVD. Templated or etched porous carbons offer more controllable pore size and connectivity, enabling tailored silicon confinement, but generally involve additional processing steps, sacrificial templates, or etching treatments, which increase fabrication complexity and cost. Graphite-based scaffolds provide high electrical conductivity and mechanical stability, but their relatively compact structure can limit internal Si infiltration. Hierarchical porous carbon/graphite frameworks can improve pore accessibility by combining different pore length scales, although excessive porosity may reduce tap density and volumetric energy density. Thus, an appropriate SiDPC scaffold should balance pore accessibility and connectivity with structural robustness, electrical conductivity, Si loading, and volumetric utilization, rather than simply maximizing specific surface area or porosity.

The enhanced structural stability is further translated into superior electrochemical performance. G14-reported laboratory-scale full cells tests delivered ∼1000 mAh g^–1^ and stable cycling over 500 cycles, which could be further extended to 1000 cycles after prelithiation.^53^ The corresponding full cells achieved ∼30% higher volumetric energy density than graphite-based counterparts. These results were obtained from G14-reported laboratory-scale evaluations and should therefore be distinguished from subsequent third-party validation. Independent third-party testing of kilogram-scale, continuously manufactured SiDPC, as reported in the corresponding technical report, demonstrated high reversible capacity, high initial Coulombic efficiency (ICE), and good consistency among replicate cells (Figure 2b).^53^ Lower specific surface area further improved ICE by suppressing irreversible lithium loss.

The most compelling advantage of this strategy lies in its engineering scalability. By integrating a rotary furnace with CVD, SiDPC can be readily scaled up from laboratory-scale gram-level synthesis to pilot-scale kilogram-level production. Its high compatibility with existing graphite anode manufacturing lines eliminates the need for extensive modifications to current battery manufacturing facilities, thereby reducing barriers to industrial implementation. At the system level, G14 further reported a BatPaC-based cost projection of below US$125 kWh^–1^.^53^ This value represents a model-based battery-pack cost projection rather than an experimentally demonstrated manufacturing cost or commercial battery price. The calculation assumed specific Si–C capacity and material-cost inputs and incorporated projected raw-material, labor, electricity, process-yield, and equipment-depreciation costs.

## 3 Structural Advantages of SiDPC: From Passive Buffering to Active Regulation

In Si–C composites, porous carbon functions not merely as a conductive or buffering matrix but, more fundamentally, as a 3D framework that provides spatial confinement.^54^ Typical porous carbons possess a high specific surface area, hierarchical pore structure, and interconnected conductive network, enabling preferential silicon growth within pores or on pore walls during CVD, rather than uncontrolled accumulation on the external particle surface. This confined deposition behavior yields a highly dispersed nanoscale silicon distribution and intimate interfacial contact with the carbon framework, fundamentally distinguishing it from the particle-level mixing characteristic of conventional mechanically blended Si–C composites.^55^ In SiDPC, nanoscale silicon is embedded within the 3D porous carbon network while preserving sufficient pore volume to accommodate volume expansion. This architecture fundamentally regulates silicon-anode behavior across three progressively coupled levels: (i) spatial accommodation of volume expansion, (ii) homogenization of stress distribution, and (iii) reconstruction of electron/Li^+^ transport pathways.

### 3.1 Spatial Accommodation of Volume Expansion

The principal advantage of the confined silicon deposition within SiDPC architecture lies in redirecting the pathway of silicon volume expansion (Figure 3a,b).^26,56^ Unlike conventional Si–C composite anodes, where silicon particles predominantly expand outward, leading to particle enlargement and electrode thickening, silicon in the SiDPC architecture is preferentially deposited within the porous carbon framework, with the pre-existing pore volume serving as a buffer during lithiation. Upon silicon expansion, the volume change is first accommodated by the internal pore space rather than directly driving particle enlargement,^40^ thereby transforming the conventional particle expansion–electrode thickening process into an internal pore occupation–structural reconstruction mechanism, effectively suppressing the propagation of volume change from the particle to the electrode scale. It should be noted that particle-level Si expansion does not necessarily translate directly into proportional macroscopic electrode swelling, particularly in highly porous architectures. The available pore volume can accommodate part of the local Si expansion. However, insufficient pore space or excessive Si loading may still lead to electrode-level swelling.

**Figure 3:**
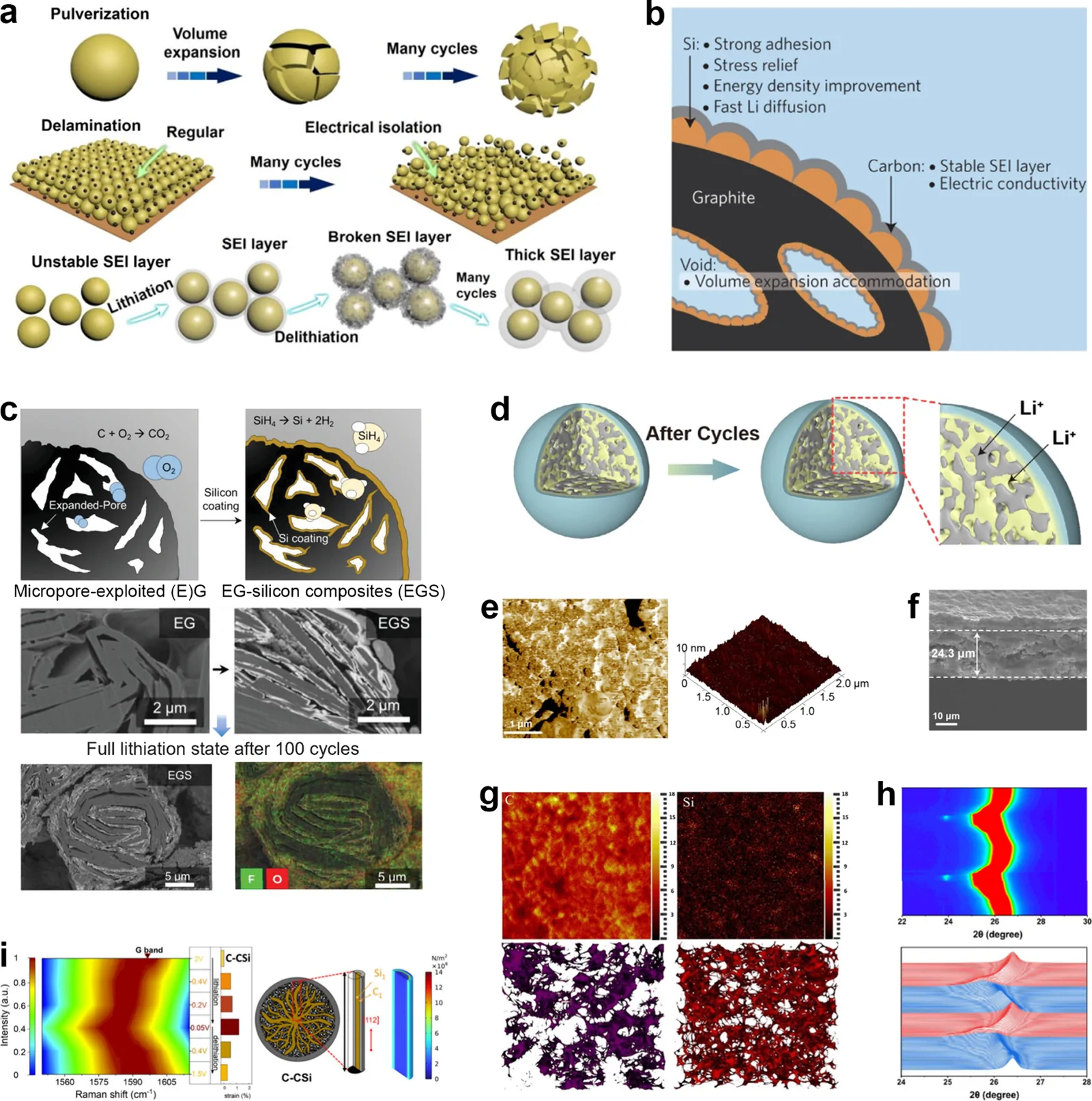
(a) Failure mechanism of silicon anode during charge and discharge process. Reproduced with permission from ref 56. Copyright 2026 Elsevier. (b) Cross-sectional schematic view showing the detailed structural characteristics of a SGC hybrid particle. Reproduced with permission from ref 26. Copyright 2016 Springer Nature. (c) Schematic illustration, microstructure, and structural evolution of Si deposition on macroporous graphite. Reproduced with permission from ref 36. Copyright 2020 Wiley. (d) Schematic diagrams of particle changes during lithiation and the Li^+^ channels. Modulus with morphology (e) and cross-sectional SEM image (f) of the electrode after 250 cycles. Reproduced with permission from ref 29. Copyright 2026 American Chemical Society. (g) TOF-SIMS chemical mapping and 3D reconstruction of carbon and silicon distributions in Si/AC0.68. Reproduced with permission from ref 51. Copyright 2025 Royal Society of Chemistry. (h) In situ XRD patterns during the discharge–charge process. Reproduced with permission from ref 60. Copyright 2025 Elsevier. (i) Lithiation and delithiation operando Raman spectra and finite simulation at a fully lithiated state. Reproduced with permission from ref 61. Copyright 2025 American Chemical Society.

From a structural design perspective, the porous carbon framework functions as a pre-engineered internal volume reservoir for silicon.^57^ This architecture substantially mitigates particle expansion while maintaining high silicon utilization, thereby enhancing dimensional stability and providing robust structural support for high-loading silicon anodes. This spatial accommodation mechanism has been corroborated by diverse SiDPC architectures employing complementary structural regulation strategies. A representative approach is the integration of macroporous graphite with CVD,^36^ which directs silicon deposition into enlarged pore channels and enables preferential inward expansion during lithiation, thereby suppressing electrode swelling while preserving interparticle electrical contact (Figure 3c). An alternative strategy reinforces the confined architecture by in situ converting surface silicon into a mechanically robust Li_*x*_SiO_*y*_ interfacial layer,^29^ which further mitigates electrode thickening and particle fracture through enhanced mechanical constraint (Figure 3d, e).

Direct structural characterization provides complementary evidence for the structural stability of electrode materials during cycling.^58,59^ Time-of-flight secondary ion mass spectrometry (TOF-SIMS) 2D elemental mapping, depth profiling, and 3D reconstruction reveal a highly uniform silicon distribution throughout the porous framework,^51^ supporting effective spatial confinement of silicon within the carbon scaffold (Figure 3g). However, such compositional mapping does not directly establish homogeneous volume accommodation during lithiation. Operando characterization provides further insight into the structural response. In situ X-ray diffraction (XRD) shows reversible lattice variation of the porous carbon framework,^60^ suggesting a largely reversible structural response during cycling rather than irreversible framework collapse (Figure 3h). Complementarily, in situ Raman spectroscopy combined with finite-element simulations indicates that the continuous interpenetrating Si–C network can distribute lithiation-induced deformation more uniformly throughout the framework,^61^ supporting the structural integrity of the composite during cycling (Figure 3i).

Spatial accommodation capability is governed not simply by porosity, but by the pore size, pore connectivity, and silicon distribution within the pore network. Insufficient porosity provides inadequate buffering space, whereas excessive porosity compromises tap density and volumetric energy density. Therefore, current SiDPC design should extend beyond increasing porosity alone toward the synergistic optimization of pore architecture and silicon deposition behavior to balance volume-expansion buffering with high energy density.

### 3.2 Homogenization of Stress Distribution

Although spatial accommodation effectively suppresses macroscopic electrode swelling, substantial internal stress remains inevitable during lithiation owing to the large volumetric mismatch of silicon. In conventional Si–C composites, where silicon exists as discrete particles,^62^ deformation is highly localized, generating pronounced stress gradients that drive crack initiation, particle pulverization, and loss of electrical contact.^63^ Therefore, mitigating stress localization and promoting its homogeneous redistribution are critical to preserving the structural integrity of silicon anodes.

Unlike pore-mediated volume accommodation, stress regulation in SiDPC originates from the continuous mechanical coupling between the confined silicon phase and the surrounding carbon framework.^64^ Rather than accumulating around individual silicon domains, lithiation-induced stress is progressively redistributed throughout the 3D interconnected network, where the continuous carbon scaffold serves as an efficient load-transfer pathway. This process enables effective stress redistribution and stress dissipation, thereby reducing peak stress, suppressing strain localization, and mitigating crack initiation.

This stress-regulation mechanism has been substantiated by complementary experimental and computational studies. A representative strategy employs a bicontinuous framework to confine silicon within a continuous carbon matrix, enabling subnanometer-scale silicon growth and facilitating uniform stress transfer and dissipation along the rigid skeleton,^24^ thereby reducing local stress concentration and inhibiting crack propagation (Figure 4a). The stress-regulating effect becomes even more pronounced in continuous Si–C interpenetrating architectures. Finite-element simulations of stress evolution at different states of charge reveal that a 3D interconnected framework composed of vertically aligned graphene and porous carbon fibers markedly suppresses stress localization, with a maximum von Mises stress of only 11.2 MPa,^65^ substantially lower than those of the reference architectures (Figure 4b,c). Further evidence comes from the combination of in situ transmission electron microscopy (TEM) and mechanical simulations. Ultrasmall silicon nanodots confined within metal–organic framework-derived porous carbon exhibit only ∼2.76% reversible dimensional variation during cycling while significantly reducing both radial and circumferential tensile stresses,^66^ thereby approaching near-zero-strain lithiation behavior (Figure 4d–f).

**Figure 4:**
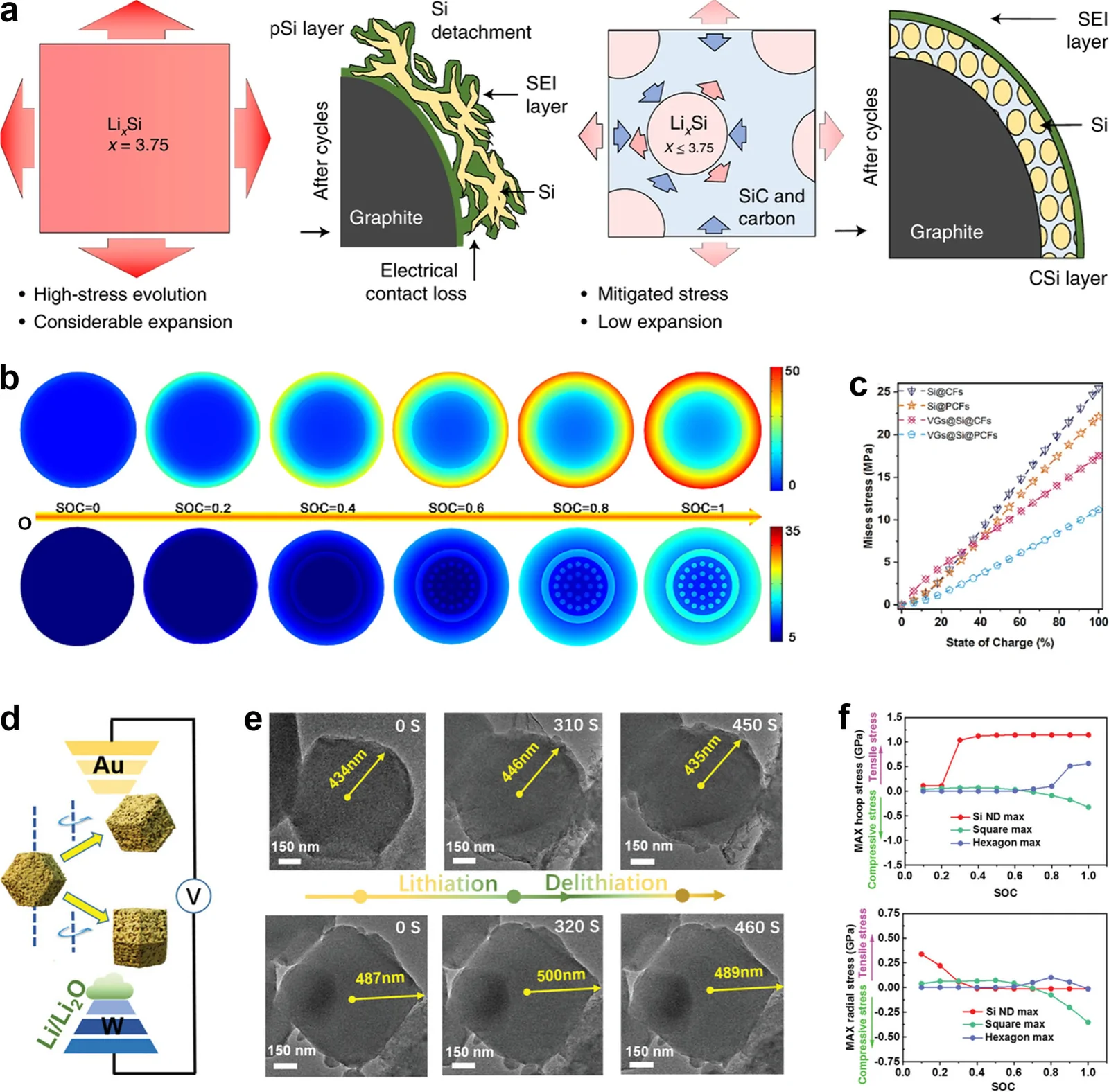
(a) Schematic illustration of the behavior of lithium silicide and corresponding fading mechanisms. Reproduced with permission from ref 24. Copyright 2021 Springer Nature. (b) Stress distributions during the lithiation process. (c) Von Mises stress in different Si-based electrodes as a function of the SOC. Reproduced with permission from ref 65. Copyright 2023 Elsevier. In situ TEM setup (d) and time-lapse TEM image (e) for individual silicon samples during a lithiation/delithiation cycle. (f) Evolution of maximum hoop stress and radial stress at different SOC values. Reproduced with permission from ref 66. Copyright 2022 Wiley.

The effectiveness of stress regulation is primarily governed by the mechanical continuity of the carbon framework, interfacial adhesion, and the efficiency of load transfer across the Si–C interface. Optimizing these factors enables more uniform stress redistribution, minimizes residual stress accumulation, and preserves structural integrity during repeated cycling.

### 3.3 Reconstruction of Electron/Li^+^ Transport Pathways

Beyond regulating volume expansion and stress evolution, SiDPC fundamentally reconstructs the transport kinetics within silicon anodes by establishing integrated pathways for electron and Li^+^ transport. In conventional mechanically mixed Si–C composites, electron conduction relies on stochastic interparticle contacts, whereas Li^+^ diffusion proceeds through tortuous particle-packed pathways to reach electrochemically active sites.^67,68^ Continuous structural evolution during cycling progressively disrupts conductive networks, increases ionic transport tortuosity, and aggravates interfacial polarization.^69^

By contrast, SiDPC enables coupled electron/Li^+^ transport throughout the electrode. Continuous carbon networks provide efficient electron conduction, while interconnected pore channels facilitate rapid Li^+^ diffusion.^60^ This integrated transport architecture reduces electronic resistance, decreases ionic tortuosity, and alleviates concentration polarization, thereby accelerating electrochemical reaction kinetics.

This transport mechanism has been validated by diverse SiDPC architectures employing complementary structural designs. A representative example is the gradient-confined CVD strategy,^70^ which uniformly deposits amorphous silicon within a microporous carbon framework to establish continuous electron-conduction networks and homogeneous Li^+^ diffusion pathways (Figure 5a–c). Electrochemical kinetic analyses reveal an increased surface-controlled capacitive contribution and accelerated Li^+^ diffusion, while finite-element simulations reveal more homogeneous Li^+^ concentration distribution and mitigated concentration polarization, corroborating improved ionic transport. A complementary strategy employs radially vertical graphene to integrate continuous electron-conduction networks with radial Li^+^ transport pathways around silicon (Figure 5d–f).^71^ Operando electrochemical impedance spectroscopy (EIS) indicates persistently low charge-transfer resistance throughout cycling, whereas first-principles calculations reveal a substantially reduced Li^+^ diffusion barrier, facilitating rapid Li^+^ migration and interfacial charge-transfer kinetics.

**Figure 5:**
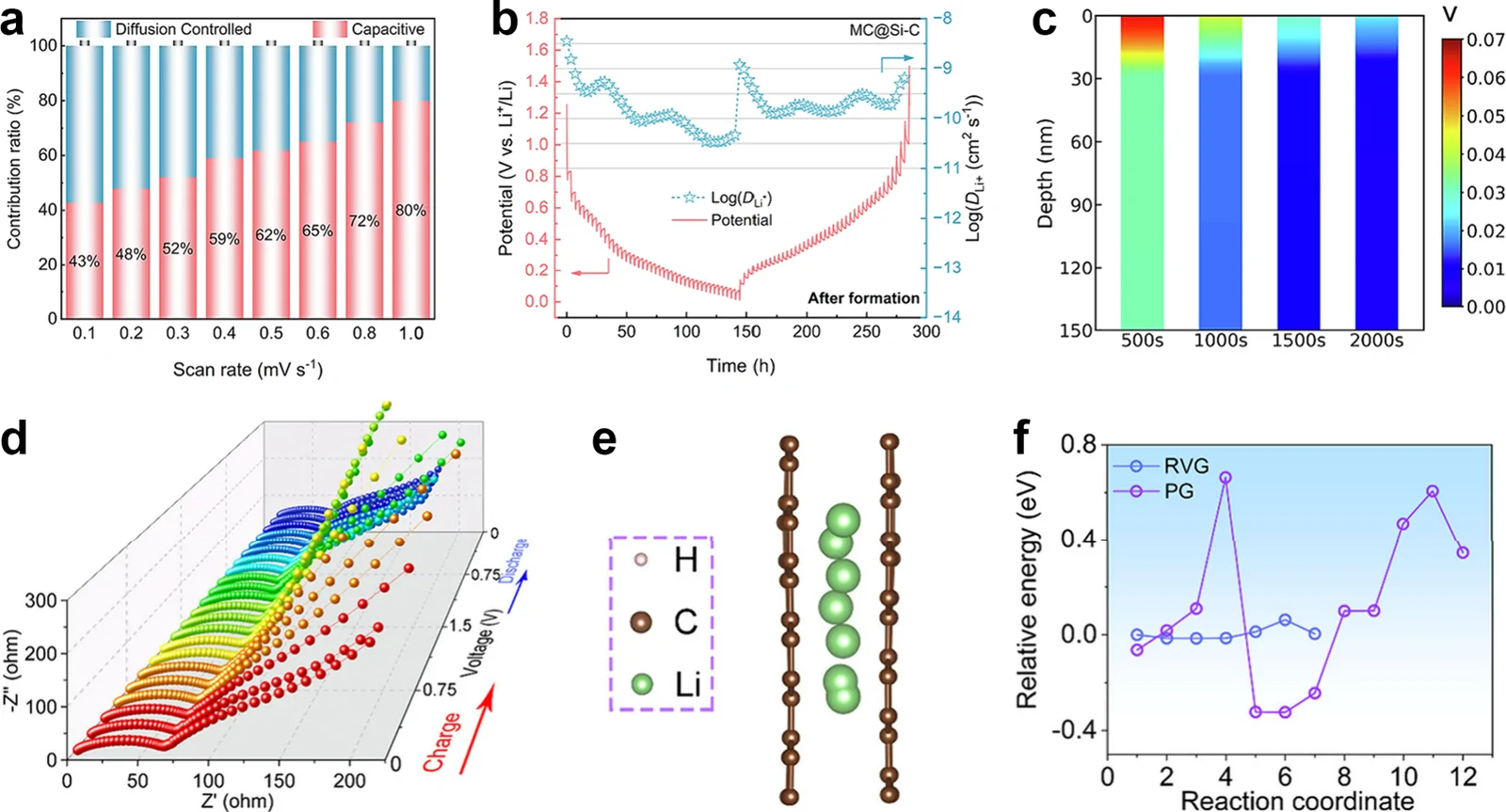
(a) Capacitive and diffusion-controlled contributions at different scan rates. (b) Li^+^ diffusion coefficients. (c) Simulated concentration-polarization potential distributions. Reproduced with permission from ref 70. Copyright 2026 Elsevier. (d) Operando EIS characterization. (e) Li^+^ migration paths in different models. (f) Diffusion barrier of Li^+^ in different models. Reproduced with permission from ref 71. Copyright 2025 Wiley.

Transport performance is governed not simply by porosity, but by the synergistic optimization of ionic and electronic transport pathways.^72^ Excessive porosity promotes electrolyte infiltration and Li^+^ diffusion at the expense of tap density and volumetric energy density, whereas insufficient pore connectivity limits ion transport and deteriorates reaction kinetics. Therefore, SiDPC design requires the coordinated optimization of transport efficiency and electrode packing density to balance rapid electron/Li^+^ transport with high volumetric energy density.

## 4 Reconstruction of the SEI Formation Behavior

Beyond mitigating volume expansion, regulating stress evolution, and optimizing transport kinetics, the porous carbon framework fundamentally reconstructs the interfacial reaction behavior of silicon anodes. In conventional silicon anodes, the SEI forms directly on exposed silicon surfaces and repeatedly undergoes fracture and regeneration during lithiation/delithiation.^73,74^ The continuous exposure of fresh silicon sustains interfacial side reactions, resulting in persistent electrolyte and active lithium consumption, reduced ICE, increased interfacial resistance, and accelerated capacity degradation.^30,75^ Although mechanically blended Si–C composites partially buffer silicon expansion, repeated deformation of discrete silicon domains continues to disrupt the SEI, leaving the fundamental challenge of interfacial instability largely unresolved.

In contrast, SiDPC fundamentally alters the spatial location of SEI formation. Because silicon is confined within the internal pore network whereas the particle exterior is dominated by a continuous carbon phase, initial SEI formation preferentially occurs on the outer carbon surface rather than directly covering the embedded silicon.^76^ Consequently, electrolyte access to internal silicon is substantially restricted, spatially confining interfacial reactions to the particle exterior instead of the entire silicon phase. However, the extent of this spatial confinement depends strongly on pore accessibility. Closed or size-selective pores can restrict electrolyte penetration and thereby limit internal SEI formation, whereas electrolyte-accessible mesopores may still permit electrolyte infiltration and subsequent SEI formation on internal silicon surfaces. Thus, pore architecture regulates not only the location but also the extent of SEI formation within SiDPC. This transition redefines the interfacial reaction field from global participation to spatially confined interfacial reactions.

In addition to pore-structure regulation, an external carbon coating can further modify the surface and interfacial behavior of SiDPC anodes. The carbon coating reduces the exposed and electrolyte-accessible surface area of silicon, thereby decreasing direct Si–electrolyte contact and suppressing parasitic reactions and irreversible lithium consumption. At the same time, the conductive carbon layer provides an additional electronic conduction pathway and helps maintain electrical contact during repeated lithiation/delithiation. By physically separating silicon from the electrolyte, an intact and uniform carbon coating can also reduce continuous electrolyte decomposition and contribute to the formation of a more stable SEI. However, these benefits strongly depend on the coating thickness, uniformity, and structural integrity. An excessively thick coating may increase Li^+^ transport resistance and reduce the accessible active surface, whereas incomplete or defective coverage can expose silicon and accelerate interfacial degradation. Therefore, carbon coating should be optimized together with the underlying pore architecture rather than treated as an independent protective layer.

This reconstructed interfacial architecture can be further reinforced through both surface and pore-structure engineering. A representative example is the introduction of sieving pores, in which subnanometer pore entrances restrict solvent penetration while preserving internal buffering space (Figure 6a,b).^23^ The resulting predesolvation of Li^+^ modifies the local solvation environment within the pore network and promotes the formation of an inorganic-rich, LiF-dominated SEI instead of a conventional organic-rich interphase. X-ray photoelectron spectroscopy depth profiling confirms that the pore architecture remains largely intact after cycling, while the SEI is spatially confined and enriched with inorganic components (Figure 6c,d).^23^ These observations indicate that pore engineering regulates not only the location of SEI formation but also its chemical composition and spatial distribution.

**Figure 6:**
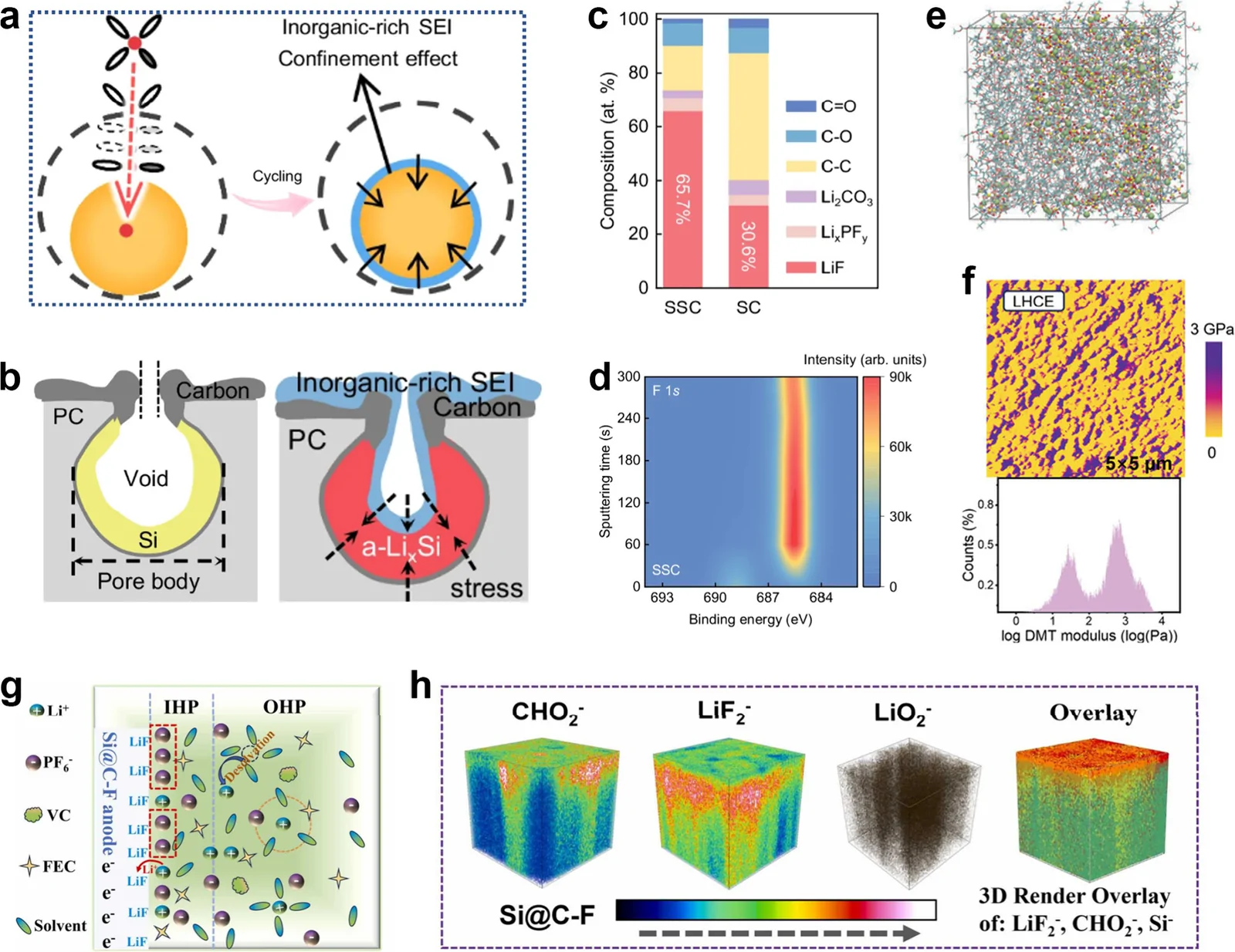
(a) Sieving-pore engineering for low-expansion silicon composite anodes. (b) Schematic illustrations of the customized microporous structure before cycling (left) and the SEI distribution within the nanopores after cycling (right). (c) Content of SEI compositions at a sputtering time of 300 s in SC and SSC negative electrodes. (d) Contour maps of F 1 s intensity for SSC negative electrodes under increasing sputtering time. Reproduced with permission from ref 23. Copyright 2025 Springer Nature. (e) Molecular dynamics analysis of electrolyte solvation. (f) LHCE profiles. Reproduced with permission from ref 77. Copyright 2024 Wiley. (g) Schematic diagram of the EDL structure corresponding to Si@C–F anode. (h) 3D TOF-SIMS mapping of SEI components. Reproduced with permission from ref 78. Copyright 2025 Elsevier.

The reconstructed interfacial architecture also provides a favorable platform for subsequent SEI engineering. On the electrolyte side, localized high-concentration electrolytes (LHCEs) regulate interfacial reactions by facilitating Li^+^ desolvation and promoting the formation of mechanically robust LiF-rich SEI (Figure 6e,f).^77^ On the electrode side, surface modification with CF_*x*_ reconstructs the inner Helmholtz plane, thereby regulating the specific adsorption of Li^+^ and fluorinated species at the electrode/electrolyte interface (Figure 6g).^78^ This interfacial environment preferentially induces the decomposition of PF_6_^–^ and fluoroethylene carbonate, leading to the formation of a uniform LiF-rich SEI.^79^ TOF-SIMS 3D reconstruction further confirms a more continuous and homogeneous inorganic interphase with reduced organic byproducts following CF_*x*_ modification (Figure 6h).

The effectiveness of interfacial stabilization depends not only on restricting electrolyte access to silicon but also on synergistically regulating pore architecture, local solvation chemistry, and interfacial reaction pathways. Future SiDPC design should therefore integrate structural confinement with electrolyte and interface engineering to simultaneously optimize SEI uniformity, chemical stability, and long-term cycling durability.

## 5 Bridging the Gap from Laboratory to GWh-Scale Manufacturing

Although SiDPC architectures have demonstrated compelling advantages in mitigating volume expansion, regulating stress evolution, optimizing transport kinetics, and reconstructing interfacial reactions,^80^ their translation into GWh-scale manufacturing remains constrained by several intrinsic trade-offs. These challenges arise not from the structural concept itself but from the difficulty of simultaneously achieving structural precision, manufacturing efficiency, and electrode-level practicality. In particular, CVD-based SiDPC must address the coupled challenges of deposition throughput, precursor utilization, thermal budget, pore-scale transport, and electrode densification during scale-up. Three interrelated issues currently dominate this transition: deposition uniformity versus manufacturing cost, porosity versus packing density, and particle-level optimization versus electrode-level engineering (Figure 7).

**Figure 7:**
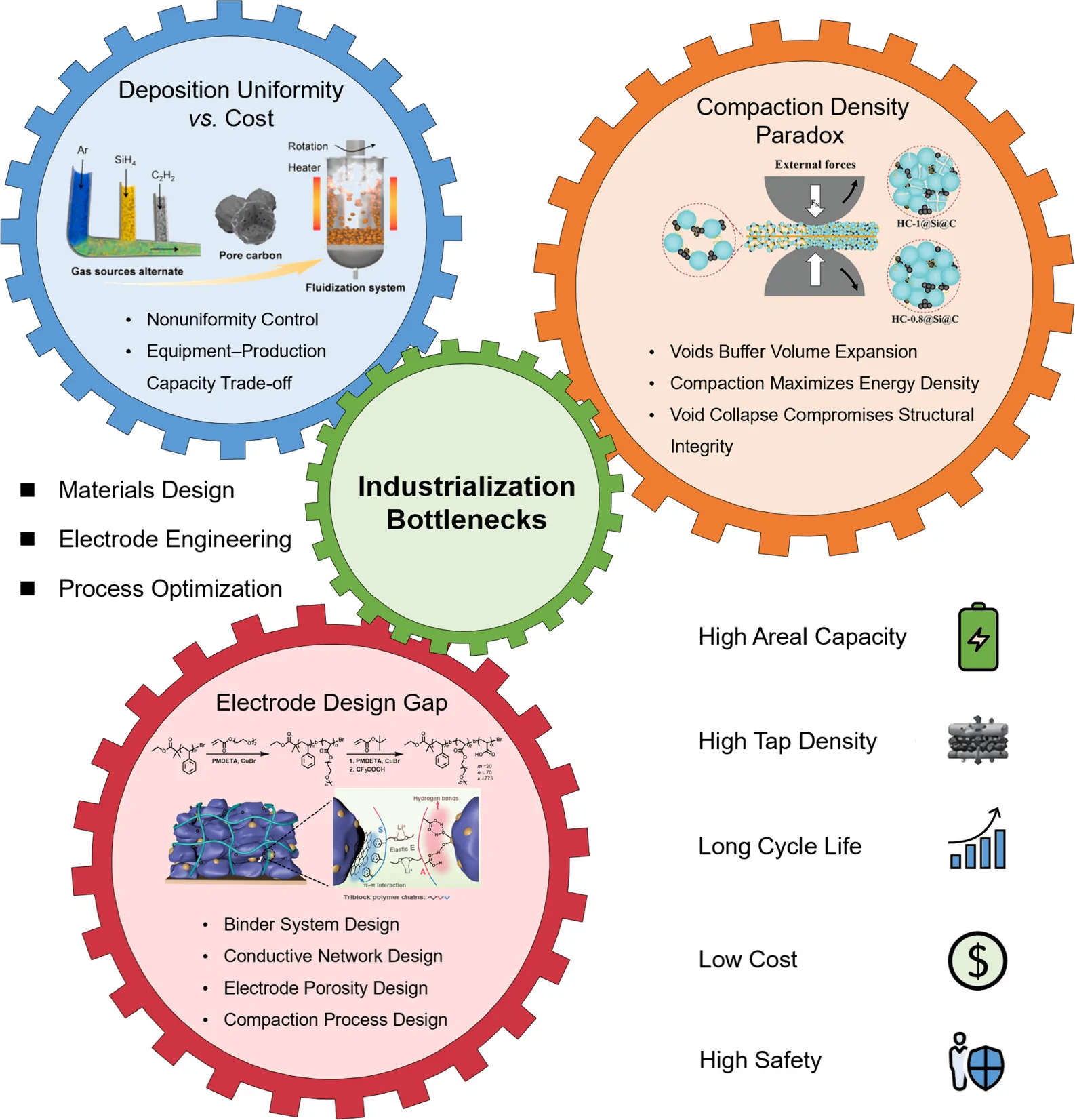
Challenges in scalable manufacturing. Reproduced with permission from ref 54. Copyright 2025 Elsevier. Reproduced with permission from ref 32. Copyright 2024 Wiley. Reproduced with permission from ref 87. Copyright 2022 Wiley.

### 5.1 Deposition Uniformity versus Manufacturing Cost

Among the available fabrication routes, CVD offers the highest degree of structural control by enabling conformal silicon deposition throughout porous carbon frameworks.^81^ In contrast, alternative low-cost synthesis strategies generally provide less precise control over silicon distribution because of nonuniform silicon incorporation, side reactions, and partial structural collapse. Consequently, current fabrication strategies are characterized by an inherent tradeoff between structural uniformity and manufacturing cost (Figure 7).^54^

For CVD-based approaches, the primary challenge lies in balancing deposition uniformity with production throughput. Uniform silicon deposition requires sufficient precursor diffusion and homogeneous nucleation throughout the internal pore network, inevitably increasing reaction time, energy consumption, and precursor usage.^82^ Moreover, the utilization efficiency of gaseous precursors can be limited by incomplete reaction and exhaust losses, further increasing material cost. Conversely, increasing deposition rate enhances productivity but promotes preferential silicon growth near particle surfaces, leading to concentration gradients, incomplete pore infiltration, and nonuniform silicon distribution. This tradeoff is particularly important for SiDPC because increasing precursor concentration or reaction temperature can accelerate deposition but may also promote external Si accumulation and pore blockage, thereby compromising the very confinement effect that distinguishes SiDPC from conventional Si–C composites.

Several strategies could therefore be pursued to improve the scalability of CVD-derived SiDPC. First, precursor utilization can be improved through optimized gas-flow patterns, precursor recycling, and staged precursor feeding, thereby reducing unreacted precursor loss. Second, reactor configurations with enhanced gas–solid mixing, such as fluidized-bed and continuous-flow reactors, can improve mass transfer and deposition uniformity while increasing throughput. Third, spatially or temporally controlled precursor concentration and temperature profiles could enable faster deposition without sacrificing penetration into the internal pore network. Importantly, lowering the deposition temperature or adopting localized/rapid heating strategies could reduce the thermal budget and minimize thermally induced evolution of the porous carbon framework.

Recent advances in reactor engineering have partially alleviated this conflict. Fluidized-bed and other continuous CVD reactors improve gas–solid mixing and mass transfer,^39^ thereby reducing concentration gradients and providing a more homogeneous deposition environment than conventional fixed-bed reactors. These configurations have already been adopted in industrial-scale production by leading silicon-anode manufacturers. Nevertheless, reactor optimization primarily mitigates rather than eliminates the intrinsic tradeoff between deposition uniformity and manufacturing cost. In addition, CVD requires precise control of gas flow, temperature, pressure, and reaction time, which increases process complexity and capital/operational requirements during scale-up. The high-temperature operation also contributes to substantial energy consumption. Future process development should therefore improve gas utilization, deposition efficiency, and reactor productivity while maintaining homogeneous silicon infiltration throughout the porous framework. Future process development should therefore move beyond simply increasing deposition rate toward improving the overall deposition efficiency, defined by the coupled optimization of precursor utilization, Si deposition yield, reactor throughput, energy consumption, and structural uniformity.

The porous carbon framework itself also governs deposition behavior. Pore size, pore connectivity, and accessible surface area collectively determine precursor transport and silicon nucleation within the pore network.^51^ Excessively small pores are susceptible to pore blockage, whereas oversized pores weaken confinement and reduce volumetric utilization. Consequently, carbon frameworks must be designed by simultaneously considering structural robustness, pore accessibility, precursor transport, and manufacturing cost.

### 5.2 Porosity versus Packing Density

The pore architecture that underpins the electrochemical stability of SiDPC simultaneously limits its practical volumetric energy density.^83^ Although internal pores accommodate silicon expansion and preserve structural integrity, they also reduce tap density and packing density,^32^ creating an intrinsic conflict between pore functionality and electrode densification (Figure 7).

This tradeoff becomes particularly evident during electrode manufacturing. High calendering pressure is essential for achieving the packing density required in practical LIBs, yet excessive compression may irreversibly collapse the pore architecture, eliminating the internal buffering space originally designed to accommodate silicon expansion.^84^ Conversely, preserving highly porous structures improves mechanical stability but sacrifices volumetric capacity under practical electrode conditions.

Accordingly, laboratory-scale materials optimized for long cycling through highly porous architectures often fail to satisfy the packing-density requirements of commercial electrodes.^85^ Recent structural designs therefore increasingly emphasize mechanically robust carbon frameworks and hierarchical pore architectures capable of retaining functional pore space under practical calendering conditions.^86^ Rather than maximizing porosity, current design strategies increasingly focus on maximizing pore functionality by separating ion-transport pathways from volume-buffering regions while maintaining structural stability during densification.

This tradeoff becomes particularly evident during electrode manufacturing. High calendering pressure is essential for achieving the packing density required in practical LIBs, yet excessive compression may irreversibly collapse the pore architecture, eliminating the internal buffering space originally designed to accommodate silicon expansion. Conversely, preserving highly porous structures improves mechanical stability but sacrifices volumetric capacity under practical electrode conditions.

Accordingly, laboratory-scale materials optimized for long cycling through highly porous architectures often fail to satisfy the packing-density requirements of commercial electrodes. Recent structural designs therefore increasingly emphasize mechanically robust carbon frameworks and hierarchical pore architectures capable of retaining functional pore space under practical calendering conditions. Rather than maximizing porosity, current design strategies increasingly focus on maximizing pore functionality, by separating ion-transport pathways from volume-buffering regions while maintaining structural stability during densification.

This balance is also critical for achieving high-rate performance. Although interconnected pores facilitate electrolyte penetration and Li^+^ transport, excessive pore volume can increase transport distances at the electrode scale and reduce the volumetric fraction of electrochemically active material. Conversely, overly compact architectures may restrict electrolyte accessibility and increase ionic transport resistance. Future SiDPC designs should therefore employ hierarchical and interconnected pore networks, in which macropores or larger transport channels facilitate rapid electrolyte/Li^+^ transport while smaller pores provide localized space for silicon confinement and volume accommodation. Such multiscale pore architectures could simultaneously improve rate capability, structural stability, and volumetric energy density.

### 5.3 Particle Optimization versus Electrode-Level Engineering

Despite substantial progress in particle-level engineering, electrode-level design remains comparatively underexplored. In practical LIBs, electrochemical performance is determined not only by particle properties but also by electrode architecture, formulation, and manufacturing processes.^88^ Consequently, optimizing individual particles does not necessarily translate into optimal electrode performance (Figure 7).^87^

Binder design exemplifies this compromise. Stronger binders improve mechanical integrity and maintain particle cohesion during repeated volume changes, yet increased rigidity often reduces electrode compressibility and limits achievable packing density. Conductive-network design presents a similar balance. 1D and 2D conductive additives,^89,90^ such as carbon nanotubes and graphene, enhance electronic percolation and network stability during cycling but increase slurry-processing complexity and manufacturing cost.

Calendering illustrates the coupling between material design and electrode manufacturing. Increasing calendering pressure improves electrode density and electronic contact, whereas excessive compression damages pore architectures and restricts ionic transport.^91^ Insufficient densification, in contrast, preserves structural integrity but compromises practical volumetric capacity. Electrode optimization therefore requires simultaneous consideration of pore preservation, electronic conductivity, ionic transport, and densification under realistic manufacturing conditions.

Importantly, the apparent advantages of SiDPC at the particle level should not be assumed to directly translate into proportional improvements at the electrode level. The internal pore volume of individual SiDPC particles can accommodate silicon expansion locally, whereas overall electrode swelling is additionally governed by particle packing, binder deformation, conductive-network rearrangement, and calendering-induced structural changes. Therefore, future evaluation should move beyond particle-level capacity retention toward electrode-level metrics, including areal capacity, electrode swelling, compacted density, volumetric energy density, and cycling stability under practically relevant mass loadings.

This distinction highlights that the transition from optimized SiDPC particles to practical electrodes requires coordinated control of particle architecture, electrode formulation, and manufacturing parameters. In particular, CVD deposition efficiency, pore accessibility, silicon distribution, electrode densification, and ion/electron transport should be optimized simultaneously rather than independently.

From this perspective, the future development of SiDPC should shift from a material-centric optimization strategy toward an integrated manufacturing strategy. At the particle level, Si confinement, pore connectivity, and Si distribution should be optimized for rapid ion transport and structural stability; at the electrode level, slurry formulation, conductive-network construction, calendering, and electrode thickness should be simultaneously adjusted to preserve these functions after densification. Such multiscale optimization is essential for converting the structural advantages observed in laboratory-scale SiDPC particles into practical improvements in areal capacity, rate capability, swelling control, and volumetric energy density.

Manufacturing reproducibility should also become a standard criterion for evaluating emerging SiDPC architectures. Although many published studies emphasize the electrochemical performance of representative electrodes, comparatively fewer systematically evaluate batch-to-batch variability or process reproducibility. Because silicon deposition within porous carbon is sensitive to pore accessibility, precursor transport, and local deposition kinetics, relatively small variations in processing conditions may lead to significant differences in silicon distribution and, consequently, electrochemical performance. Future studies should therefore report batch-to-batch reproducibility together with statistical variation in key metrics such as silicon loading, ICE, areal capacity, capacity retention, and impedance evolution.

Beyond reproducibility, practical relevance should ultimately be assessed using a standardized set of electrode- and cell-level metrics rather than gravimetric capacity alone. For emerging SiDPC architectures, this reporting set should include electrode composition (active-material fraction), calendered density, porosity, thickness, electrode swelling, ICE, average Coulombic efficiency, areal capacity, capacity retention, electrolyte-to-capacity ratio, N/P ratio, and full-cell gravimetric and volumetric energy density. ICE is particularly important because irreversible lithium consumption directly affects the lithium inventory available in the full cell, whereas average Coulombic efficiency provides a complementary measure of cumulative lithium loss during prolonged cycling. Similarly, areal capacity and electrode density provide more direct measures of practical electrode utilization and volumetric performance than gravimetric capacity alone. At the full-cell level, electrolyte amount and N/P ratio must also be considered because excessive electrolyte or imbalanced electrode capacities can substantially distort the apparent energy density and material-level advantages of SiDPC.

Accordingly, areal capacities approaching or exceeding 3 mAh cm^–2^, together with controlled electrode swelling, adequate electrode density, realistic electrolyte-to-capacity ratios, and appropriate N/P balancing, provide a more discriminating assessment of practical SiDPC performance than long-term cycling under dilute-loading half-cell conditions. For example, a hierarchical porous Si–C/graphite electrode operating at 3 mAh cm^–2^ exhibited less than 20% initial swelling and approximately 92% capacity retention over 500 cycles. Silicon-based full-cell studies further demonstrate the importance of evaluating performance at the complete-cell level rather than relying exclusively on half-cell measurements. Cell-level analyses also indicate that areal capacity, electrode composition, and cell-component dimensions strongly influence attainable gravimetric and volumetric energy density, while manufacturing-cost models emphasize the importance of cell format, chemistry, process yield, capital expenditure, and production scale when assessing commercial feasibility.

## 6 Toward Programmable Si–C Architectures

The development of SiDPC has progressively evolved from constructing porous Si–C architectures to understanding the mechanisms governing volume accommodation, stress regulation, transport kinetics, and interfacial reactions.^92^ Future advances are therefore expected to move beyond static structural optimization toward programmable Si–C architectures, in which lithiation behavior can be deliberately designed, dynamically regulated, and ultimately predicted across multiple length scales (Figure 8). Rather than simply depositing silicon within porous carbon, next-generation architectures should enable coordinated control over structural evolution and electrochemical reactions throughout battery operation.

**Figure 8:**
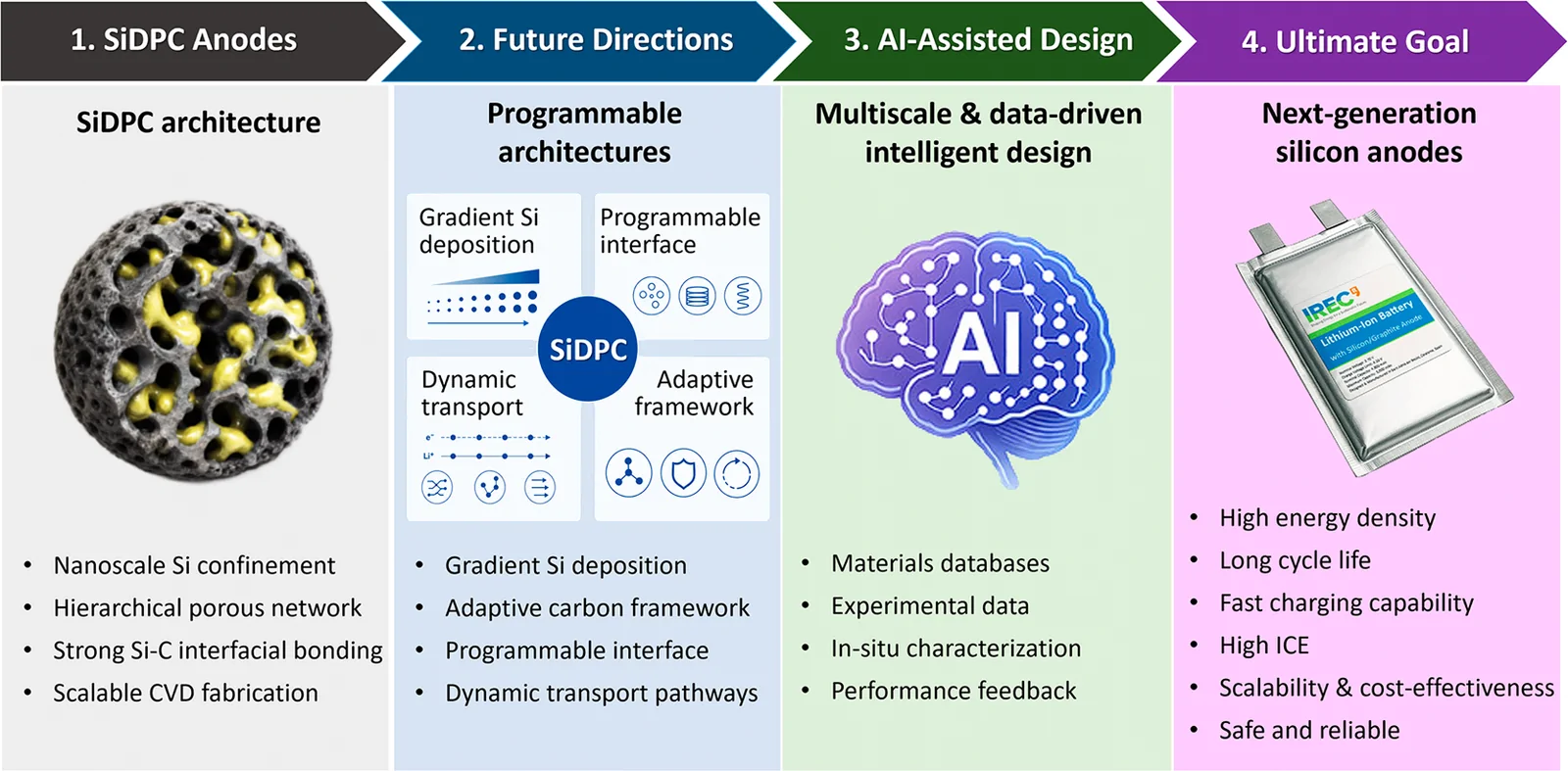
Future perspectives toward programmable SiDPC architectures. Reproduced with permission from ref 94. Copyright 2026 Wiley. Reproduced with permission from ref 95. Copyright 2025 Wiley.

A central direction toward this goal is the development of structurally programmable architectures. Gradient silicon deposition, mechanically adaptive carbon frameworks, and dynamically reconfigurable pore structures offer opportunities to regulate lithiation fronts in both space and time. Such architectures may simultaneously optimize volume accommodation, stress dissipation, active-material utilization, and transport pathways by tailoring where and how lithiation proceeds, rather than relying on static pore geometries alone (Figure 8).^93^ Future porous carbon frameworks are therefore expected to function not merely as passive hosts for silicon but as active structural regulators of electrochemical reactions.

At the design level, artificial intelligence (AI) and computational optimization are expected to fundamentally reshape the development of SiDPC architectures.^28^ To make data-driven design more applicable to SiDPC, a dedicated structure–process–property data set should be established by integrating experimentally measurable structural, processing, mechanical, and electrochemical parameters. Key structural descriptors could include pore-size distribution, pore volume and connectivity, accessible surface area, Si loading and spatial distribution, carbon-framework mechanical properties, and electrode density. CVD processing parameters, such as deposition temperature, precursor concentration, gas composition, and deposition time, could be incorporated as process-level descriptors to establish quantitative relationships between synthesis conditions and the resulting Si–C architecture. These parameters could then be correlated with electrochemical and structural outputs, including ICE, average Coulombic efficiency, reversible capacity, areal capacity, rate capability, capacity retention, electrode swelling, and volumetric energy density.

For example, pore-size distribution and connectivity describe the available space and Li^+^ transport pathways for Si accommodation, while Si loading and spatial distribution are directly related to silicon utilization and volume-expansion behavior. The mechanical properties of the carbon framework can further reflect its ability to constrain structural deformation, whereas electrode density and volumetric energy density provide practical metrics for evaluating the tradeoff between porosity, structural stability, and energy storage. Based on such a data set, machine learning could be used to identify the descriptor combinations associated with targeted performance, while topology optimization and multiscale simulation could further refine pore architecture, Si distribution, and mechanical characteristics. The optimization targets should therefore extend beyond gravimetric capacity to include high initial and average Coulombic efficiency, high areal capacity, suppressed electrode swelling, adequate electrode density, long-term cycling stability, and high full-cell gravimetric and volumetric energy density. Integration with digital twins could ultimately enable iterative prediction, experimental validation, and process optimization, making the concept of a “programmable Si–C architecture” more concrete and practically actionable.

Structural innovation alone, however, will be insufficient without simultaneous advances in interfacial chemistry and electrode manufacturing. Future research should therefore integrate particle architecture, interfacial engineering, electrode formulation, and manufacturing processes into a unified design framework. Artificial interphases, electrolyte engineering, prelithiation strategies, calendering optimization, and electrode-level structural design should no longer be considered independent research directions but rather complementary components of an integrated manufacturing strategy. Ultimately, the practical competitiveness of silicon anodes will depend not only on the intrinsic properties of individual particles, but also on how effectively these coupled processes are coordinated under commercially relevant electrode configurations.^96^

The significance of SiDPC therefore extends beyond introducing another silicon-anode architecture. More fundamentally, it establishes a structural framework in which volume accommodation, stress regulation, transport kinetics, and interfacial reactions can be engineered in a coordinated manner. Future progress will likely depend less on maximizing silicon content than on improving the precision with which these coupled processes are regulated across multiple length scales. In this context, programmable Si–C architectures may provide a rational pathway toward next-generation high-energy-density LIBs.
